# Dickkopf-related protein 2 induces G0/G1 arrest and apoptosis through suppressing Wnt/β-catenin signaling and is frequently methylated in breast cancer

**DOI:** 10.18632/oncotarget.17055

**Published:** 2017-04-12

**Authors:** Junhao Mu, Tianli Hui, Bianfei Shao, Lili Li, Zhenfang Du, Li Lu, Lin Ye, Shuman Li, Qianqian Li, Qian Xiao, Zhu Qiu, Yan Zhang, Jiangxia Fan, Guosheng Ren, Qian Tao, Tingxiu Xiang

**Affiliations:** ^1^ Chongqing Key Laboratory of Molecular Oncology and Epigenetics, The First Affiliated Hospital of Chongqing Medical University, Chongqing, China; ^2^ Cancer Epigenetics Laboratory, Department of Clinical Oncology, State Key Laboratory of Oncology in South China, Sir YK Pao Center for Cancer and Li Ka Shing Institute of Health Sciences, The Chinese University of Hong Kong and CUHK Shenzhen Research Institute, Hong Kong; ^3^ Chinese Medicine Hospital of Linyi City, Shandong, China

**Keywords:** DKK2, tumor suppressor, methylation, cancer, Wnt signaling

## Abstract

Dickkopf-related protein 2 (*DKK2*) is one of the antagonists of Wnt/β-catenin signaling, with its downregulation reported in multiple cancers. However, how *DKK2* contributes to breast tumorigenesis remains unclear. We examined its expression and promoter methylation in 10 breast tumor cell lines, 98 primary tumors, and 21 normal breast tissues. Compared with normal tissues, *DKK2* was frequently silenced in breast cell lines (7/8). *DKK2* promoter methylation was detected in 77.8% of cell lines and 86.7% of breast tumors; while rarely detected in normal breast tissues (19%), indicating common *DKK2* methylation in breast cancer. Ectopic expression of *DKK2* changed breast tumor cell morphology, inhibited cell proliferation and colony formation by inducing G0/G1 cell cycle arrest and apoptosis, and suppressed tumor cell migration by reversing epithelial-mesenchymal transition (EMT) and downregulating stem cell markers. Moreover, restored expression of *DKK2* in MCF7 cells disrupted the microtube formation of human umbilical vein endothelial cells on Matrigel®. *In vivo*, the growth of MDA-MB-231 cells in nude mice was markedly decreased after stable expression of *DKK2*. *DKK2* suppressed canonical Wnt/β-catenin signaling by inhibiting β-catenin activity with decreased active β-catenin protein. Thus, our findings demonstrate that *DKK2* functions as a tumor suppressor through inhibiting cell proliferation and inducing apoptosis via regulating Wnt signaling during breast tumorigenesis.

## INTRODUCTION

Wnt/β-catenin pathway is critical to multiple tumorigenesis [1]. Both genetic and epigenetic changes cause abnormal activation of Wnt pathway components that are involved in multiple cell functions, contributing to tumor initiation and development.

Wnt signaling is comprised of canonical Wnt/β-catenin and non-canonical Wnt signaling that are independent of β-catenin [2]. Increased expression of Wnt family members greatly increases the risk of breast tumor formation [3–5]. Moreover, a majority of breast cancer studies reveals that Wnt antagonists can be regulated by epigenetic changes. Importantly, DNA methylation is frequently detected in this pathway in multiple cancers, especially Wnt antagonists are frequently down-regulated by promoter CpG methylation, suggesting that aberrant epigenetic changes towards Wnt signaling, rather than gene deletion or mutation, are involved in breast tumorigenesis [2, 6–10].

*DKK2*, Like *DKK1, DKK3, DKK4* and *Soggy*, belong to the DKK family [11]. It locates at 4q25 and regulates Wnt/β-catenin signaling through binding to LDL receptor-related proteins (LRP5/6). *DKK1, 2* and *3*, but not *DKK4*, have typical CpG island in their promoters, thus are regulated by epigenetic mechanism via promoter CpG methylation [2]. *DKK2* has been shown to be downregulated or silenced by epigenetic mechanisms in several malignancies, including Ewing's sarcoma, kidney cancer, and ovarian cancer, same as *DKK3* [12–14]. Although *DKK2* is frequently silenced by promoter methylation, its effects on Wnt signaling in breast carcinogenesis are still unclear.

Here we investigated the expression and significance of *DKK2 in* mammary cancer, as well as its functions *in vivo* and *in vitro*. Our findings demonstrate that *DKK2* inhibited breast cancer growth through downregulating activated β-catenin levels. The tumor-specific promoter methylation of *DKK2* could be a potential marker for the early assessment of mammary cancer.

## RESULTS

### DKK2 is downregulated in breast carcinoma

**Figure 1:**
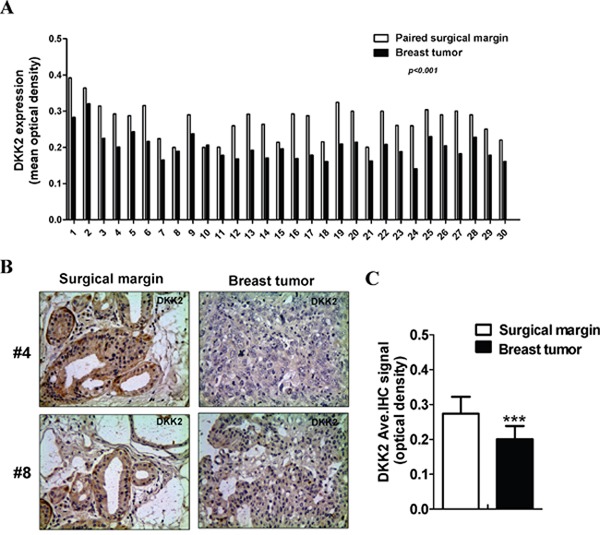
The expression levels of DKK2 in breast cancer tissues **(A)** The MOD of DKK2 protein in each case. **(B)** Representative images of DKK2 IHC staining in paired breast carcinomas and its surgical margin tissues. **(C)** Quantitative analysis of the MOD of *DKK2* expressions in two groups are shown as values of mean ± SD. ***, *p* < 0.001(Student's *t*-test).

**Figure 2:**
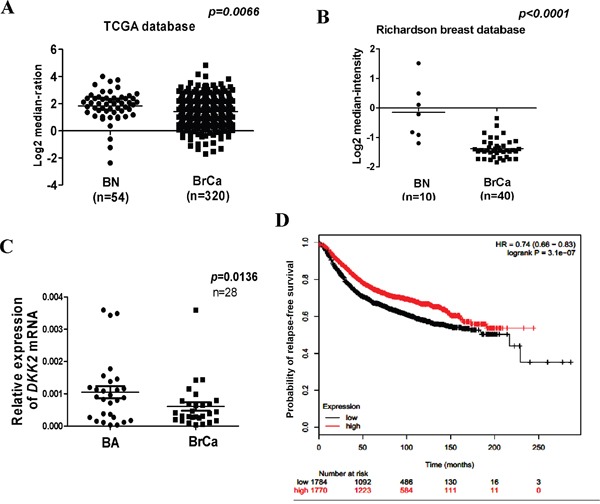
Expression and significant of *DKK2* in breast carcinoma **(A, B)** *DKK2* expression is reduced in breast carcinoma. Oncomine platform provides all data (https://www.oncomine.org/). BN: normal breast tissue; BA: breast adjacent tissue; BrCa: breast cancer tissue. **(C)** Expression of *DKK2* was detected by quantitative real-time PCR in human breast adjacent tissue and breast cancer tissues. **(D)** *DKK2* expression and prognostic analyses in breast carcinoma. Kaplan-Meier survival curves are presented to explicate prognostic significant of *DKK2*. Data from bc-GenExMiner. The *p* values shown was statistically significant.

*DKK2* downregulation in breast cell lines has been reported in our previous study [15]. Protein expression of DKK2 was examined in 30 paired primary tumors and appropriate surgical-margin tissues by immunohistochemistry (IHC). IPP6.0 analysis showed that DKK2 protein expression was significantly lower in breast tumors (0.201 ± 0.038) than that in surgical-margins (0.274 ± 0.049) (***p < 0.001) (Figure 1A–1C). Furthermore, we showed that *DKK2* mRNA level was significantly lower in breast cancer tissues than that in paired surgical-margins by qRT-PCR (*p < 0.05) (Figure 2C). Alternatively, the downregulation of *DKK2* was related to clinicopathological subtypes of breast cancer according to data from Oncomine database (Oncomine, Compendia Bioscience, Ann Arbor, MI) (Figure 2A, 2B) (*p* < 0.00001). Altogether, these data indicated that the reduced *DKK2* expression was a solid fact in breast carcinoma.

Prognostic analyses showed that higher levels of *DKK2* expression could herald a better survival rate [hazard ratio (HR) = 0.74, *p* = 3.1 e-07] (Figure 2D) [16–17]. These results indicated that *DKK2* downregulation may be a marker to evaluate the outcome of breast carcinoma.

### Promoter CpG methylation downregulated *DKK2* expression

**Figure 3:**
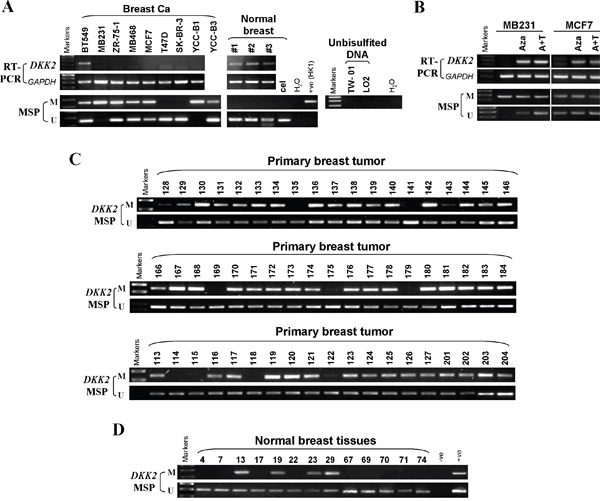
The methylation status of *DKK2* promoter in mammary carcinoma cell lines, primary tumor tissues and normal breast tissues **(A)** Promoter methylation of *DKK2 in* mammary carcinoma cells. “M” indicates methylated *DKK2*; “U” indicates unmethylated *DKK2*. **(B)** Restoration of *DKK2* expression by Aza with or TSA treatment in MDA-MB-231 and MCF7 cells. **(C)** Representative methylation of *DKK2* in mammary carcinoma and **(D)** normal tissues as measured by MSP.

*DKK2* contains a typical CpG island [15]. To identify whether *DKK2* silencing was due to its promoter CpG methylation, we investigated methylation status of *DKK2*. *DKK2* was silenced in 7/8 breast cell lines (Figure 3A), while its CpG hypermethylation was detected in 7/9 cell lines (Figure 3A). To further determine whether *DKK2* silencing correlated with promoter methylation, we treated MDA-MB-231 and MCF7 cells with demethylation drug 5-Aza-dC or combined with TSA. Results showed that *DKK2* expression was remarkably restored after treatment, together with increased unmethylated alleles and decreased methylated alleles (Figure 3B). Thus, *DKK2* silencing or downregulation was the result of promoter CpG methylation in breast cancer cells.

Furthermore, 98 primary breast carcinoma tissues and 21 normal mammary tissues were analyzed by MSP to investigate *DKK2* methylation in breast tumors. *DKK2* methylation was detected in 85/98 (86.7%) breast tumors, 4/21 (19%) in normal tissues, indicating that *DKK2* methylation was a common in breast cancer (Table 1, Figure 3C, 3D). These results suggested that the promoter of *DKK2* is specifically methylated in breast tumors.

**Table 1:** Methylation status of the *DKK2* promoter in primary breast tumors

Samples	*DKK2* promoter		Frequency of methylation
	methylation	unmethylation	
BrCa (n= 98)	83	13	86.7%
BNP (n=21)	4	21	19%

Note: BrCa, breast cancer; BNP, breast normal tissues

However, *DKK2* methylation was not statistically associated with age, tumor size, clinical stage, metastasis, or ER, PR, HER2 status of breast carcinoma patients through analyzing the *DKK2* promoter methylation and patient clinicopathological characters. All these results indicated that *DKK2* methylation is a potential marker for breast carcinoma early detection.

### *DKK2* inhibits breast cancer cell growth and colony formation

**Figure 4:**
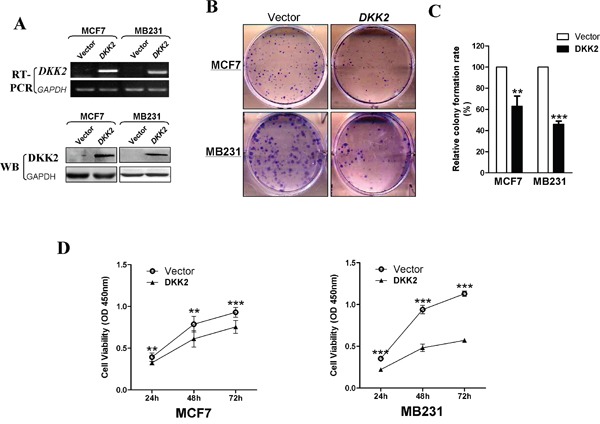
Inhibitory effect of *DKK2* in breast carcinoma **(A)** Expression of *DKK2* was confirmed by RT-PCR and western blot in vector- and *DKK2*- infected MDA-MB-231 and MCF-7 cells. **(B)** Representative images of CFA in vector- and *DKK2*-infected mammary carcinoma cells. **(C)** The histogram statistics of CFA. The mean ± S.E. data from three independent experiments (***p < 0.001). **(C) (D)** Measurement of cell proliferation for vector- and *DKK2*- infected mammary carcinoma cells through CCK-8 assay. Three asterisks, *p* < 0.001.

To explore the tumor suppressive function of *DKK2* in breast cancer, CCK8 assays and colony formation assays were performed in MDA-MB-231 and MCF7 cells. *DKK2* expression in *DKK2*-infected cells were verified via RT-PCR and western blot (Figure 4A). Compared to the control cells, there was a 40-65% reduction in colony formation in *DKK2*-expressed cells (***p < 0.001, **p < 0.01) (Figure 4B, 4C). Cell vitality, as determined by CCK8 assay, also remarkably declined at 24 h, 48 h, and 72 h (all ***p < 0.001) (Figure 4D).

### *DKK2* delays cell cycle and induces apoptosis in mammary carcinoma cells

**Figure 5:**
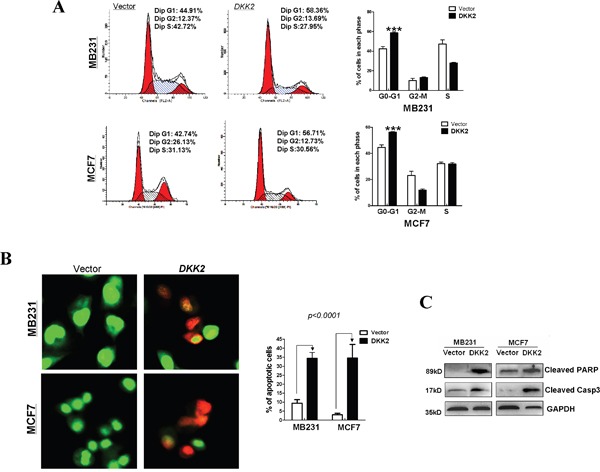
Flow cytometric analysis **(A)** The cell cycle distribution in vector and *DKK2*- infected MDA-MB-231 and MCF7 cells detected by flow cytometry analyses. Representative images of cell cycle distribution (left) and statistical graphs of cell cycle changes (right) (***p < 0.001). **(B)** AO/EB staining was used to examine cell apoptosis. The percentage of total apoptotic cells is calculated and indicated in histograms (***p < 0.001). **(C)** DKK2 upregulated the expression levels of two classic markers of apoptosis, cleaved-PARP and cleaved-caspase 3, by western blot.

Cell cycle analysis was performed to explore how *DKK2* affects cell proliferation. Compared to control cells, *DKK2* significantly increased the number of MDA-MB-231 and MCF7 cells in G0-G1 phase by 14% and 13%, respectively (***p < 0.001) (Figure 5A). Acridine orange/ethidium bromide (AO/EB) double staining was performed to examine breast tumor cell apoptosis (Figure 5B). These data indicated that *DKK2* can inhibit cell growth by delaying the cell cycle in G0/G1 and inducing cell apoptosis. Western blot further showed increased expression of cleaved-PARP and caspases in *DKK*2 expressing cells (Figure 5C).

### DKK2 inhibits breast cancer xenografts' growth in nude mice

**Figure 6:**
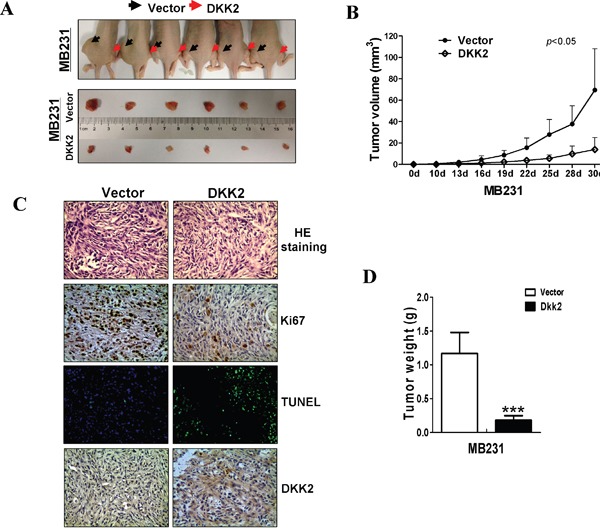
*DKK2* inhibited mammary carcinoma growth *in vivo* **(A)** Images of human breast tumor xenografts. **(B)** Comparative analyses of tumor growth curve for vector- and *DKK2-* infected MDA-MB-231 cells in nude mice xenografts (***p < 0.001). **(C)** Representative photographs of H&E staining and IHC expression analyses of *DKK2*, Ki-67, and apoptosis as assessed by TUNEL assays in xenografts (400× magnification). **(D)** Comparative histogram of tumor weights in the two groups of nude mice (***p < 0.001).

Nude mice xenografts model was used to further assess the functions of DKK2 *in vivo*. No significant differences were observed in the various organs between the two group mice (data not shown). The average volume and weight of the tumors were significantly lower in DKK2 group than that in control group (***p < 0.001) (Figure 6A, 6B). IHC and hematoxylin & eosin (H&E) staining were carried out to analyze DKK2 expression and tumor features of the xenografts nude mice, respectively. TUNEL analyses and Ki-67 staining were carried out to evaluate cell apoptosis and proliferation. Most tumor cells with frequently nuclear fragmentation were observed in xenografts with *DKK2*-expressing, along with downregulation of Ki-67 and increasing of apoptotic cells (Figure 6C, 6D). All these data indicated that DKK2 inhibits breast tumorigenesis.

### *DKK2* suppresses migration and invasion via regulating epithelial- mesenchymal transition (EMT) in breast carcinoma

**Figure 7:**
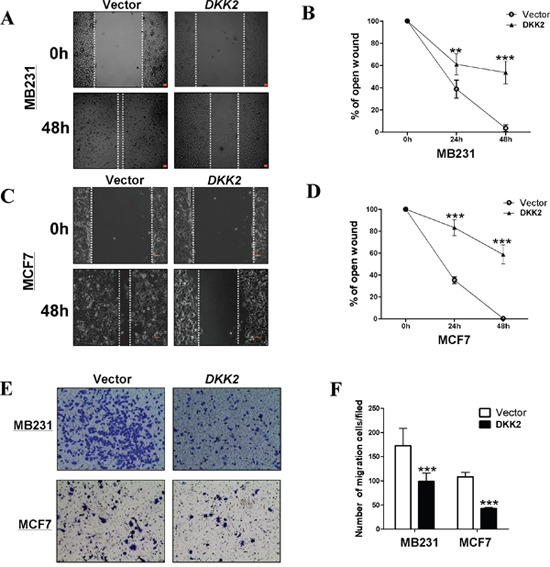
Ectopic expression of *DKK2* inhibited migration of breast cancer cells **(A, B, C, and D)** The cellular migration abilities (MDA-MB-231 and MCF7) were examined by wound healing assays. Photographs were captured at 0, 24, or 48 h. The ratio of wound healing was calculated (right). **(E, F)** *DKK2* inhibited the migration of tumor cells, 400×magnification (***p < 0.001).

To further make clear the effects of DKK2 on cell migration, wound healing assays were performed. DKK2 stable cells migrated along the wound borderline slower than the control cells at 48 h, (all ***p < 0.001) (Figure 7A-7D) indicating that DKK2 could also inhibit cell migration in MDA-MB-231 and MCF7. Transwell® assays further identified that DKK2 expressing cells had a significantly decreased number of cells through the membrane. (***p < 0.001) (Figure 7E, 7F).

**Figure 8:**
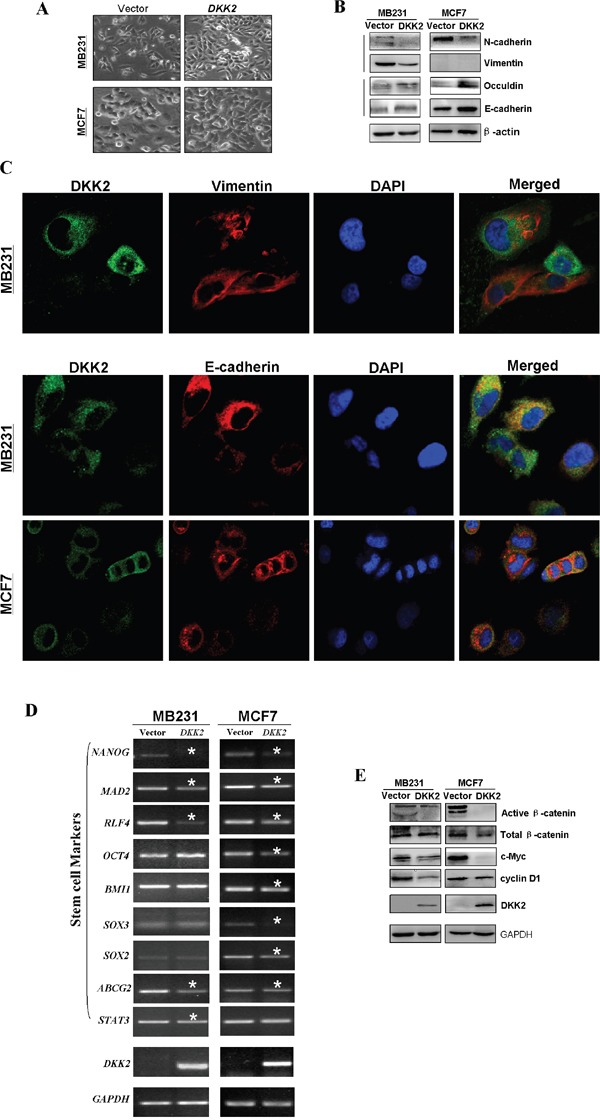
Ectopic expression of *DKK2* and the effects on EMT and Wnt signaling in breast tumor cells **(A)** Morphological changes of breast cells infected with control vector or *DKK2* by phase contrast microscopy. 400×magnification. **(B)** The thicker stress fibers of *DKK2* expressing cells and more fibers of the E-cadherin, but fewer and thinner fibers of the vimentin in *DKK2* expressing tumor cells. **(C)** Western blot analyses of EMT, and downstream target markers. **(D)** Representative stem cell markers were detected in *DKK2*-infected MDA-MB-231 and MCF7 cells by RT-PCR. *Indicates significantly decreased band density. **(E)** Examination of β-catenin and its targets by western blot.

To study underlying mechanisms of DKK2 on cell migration in breast cancer, cell morphological changes was firstly observed (Figure 8A). Cells expressing *DKK2* recovered cell adherence and contacts with each other, however, the control cells exhibited a scattering pattern, indicating that *DKK2* might be involved in tumor cell EMT. Western blot showed increased epithelial markers (E-cadherin and occludin), and decreased mesenchymal markers (N-cadherin and vimentin), in *DKK2* expressing cells (Figure 8B). Immunofluorescence staining further showed increased staining of E-cadherin and less staining of vimentin in the cytoplasm and the membranes of breast tumor cells expressing *DKK2* (Figure 8C). These results demonstrated that *DKK2* inhibits EMT in breast cancer cells.

As cell with stem cell properties is closely related to EMT for tumor cells, it is necessary to investigate whether *DKK2* could negatively regulate cell stemness in breast tumor cells. RT-PCR showed that *DKK2* caused the downregulation of most stem cell markers, including *OCT4*, *BMI1*, *SOX2*, *SOX3*, *MAD2*, *NANOG*, *ABCG2*, *STAT3*, and *RLF4 in MDA-MB-231 and MCF7* cells (Figure 8D). Again it indicated that DKK2 may inhibit both EMT and stemness of breast carcinoma cells.

### DKK2 regulates the Wnt signaling pathway in breast tumor cells

*DKK* family is an antagonist of Wnt pathway. Here we studied whether the tumor suppressive function of *DKK2* was related to its effects on this pathway. Our results showed that the restored expression of DKK2 in breast tumor cell lines significantly down-regulated active β-catenin. The downstream target genes of β-catenin, cyclin D1 and c-Myc were also confirmed to be downregulated (Figure 8E). Our findings indicated that *DKK2* inhibited β-catenin activity, thus inhibiting Wnt/β-catenin signaling in mammary cancer.

### DKK2 inhibits angiogenesis *in vitro* and in conditioned medium

**Figure 9:**
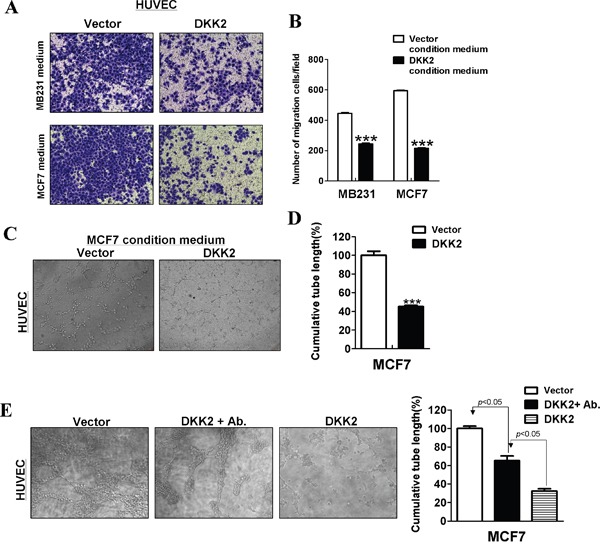
*DKK2* suppressed angiogenesis *in vitro* **(A, B)** Conditioned media from *DKK2*- infected breast tumor cells inhibited migration of HUVECs. Each experiment was performed three times. **(C, D)** Conditioned media from *DKK2*-expressing MCF7 cells suppressed HUVECs tube formation. **(E)** Primary antibodies targeting *DKK2* were used in conditioned media from *DKK2*-expressing cells. Effects of *DKK2*-expressing MCF7 cells on HUVECs tube formation were measured (***p < 0.001).

Angiogenesis plays an important role in tumor growth. To understand whether attenuating new blood vessel formation contributes to tumor suppressive function of *DKK2*, Transwell^®^ assays for HUVECs in conditioned medium were performed to verify the effects of *DKK2* on the migration of HUVECs, when compared to control cells. HUVECs cell lines cultured in DKK2 conditional medium demonstrated a significantly decreased number of migrating cells compare with the empty vector group (***p < 0.001) (Figure 9A, 9B).

Tube formation assays were further performed *in vitro*. As shown, *DKK2* significantly disrupted microtube formation of HUVECs on Matrigel^®^ (***p < 0.001) (Figure 9C, 9D). After incubation with DKK2 primary antibodies in conditioned media, DKK2 antibody could partly reverse microtubule formation of HUVECs (***p < 0.001) (Figure 9E).

## DISCUSSION

At present, a large amount of studies reported that Wnt signaling was important in initiation and development of breast carcinoma. Several Wnt/β-catenin antagonists were epigenetically repressed in breast cancer [1], including *WIF1* [18], *SFRP1* [19], *SFRP2* [20], *SFRP5* [21], *DKK1* [22], and *DKK3* [15]. Within the *DKK* family, the *DKK2* gene is a critical gene involved in the inhibition of Wnt signaling. Recently, silencing of *DKK2* was reported to be a valuable biomarker for multiple malignancies [12–14], but its roles in breast cancer are still unclear. Thus, defining the underlying mechanisms of *DKK2* in breast cancer development may support the idea that DKK2 may be a potential treatment target and marker in breast carcinoma. Here, *DKK2* promoter methylation was detected in 77.8% (7/9) of breast cell lines and in 86.7% of breast tumor samples. No methylation was observed in SK-BR-3 and T47D cells with *DKK2* downregulation, which might be caused by the heterogeneity of tumor cells, and suggests that other mechanisms such as histone deacetylation might be also involved. Our data showed that the methylation status and expression of *DKK2* were not correlated with the clinical features of breast tumor, such as pathological grade, ER status, and lymph node metastasis level. However, *DKK2* can be a new biomarker for early assessment of breast tumor.

Our results also showed that ectopic expression of *DKK2* can induce apoptosis, suppress cell growth, and halt cell cycle in G0/G1 in breast tumor cell lines, in agreement with previous reports [12–14]. *DKK2* also suppressed cell migration by interfering with EMT. Moreover, we showed that *DKK2* suppressed β-catenin activation and expression of its downstream genes. Numerous studies have demonstrated WNT pathway plays diverse roles in regulating the vascular system [23–25]. Indeed, both Wnt1 and Wnt5a have been shown to regulate two critical processes for angiogenesis, endothelial cell (EC) proliferation and migration [26]. Actually, the anti-angiogenic activities of other Wnt pathway antagonists also were reported in some studies, including sFRPs, WIF1, DKK1 and DKK4 [27–29]. On the other hand, it is interesting that in some cases Wnts seem to be inhibitory of angiogenesis, WNT1 and WNT5 were found to inhibit proliferation of endothelial cells [30]. Previous studies showed that *DKK2* boosts angiogenesis and tissue recovery by regulating the activation of Cdc42 in rodent and EC cells [31]. Our results demonstrated that *DKK2* dramatically suppresses angiogenesis compared with controls in cultured human endothelial cells. After incubating with DKK2 primary antibodies in conditioned media, DKK2 antibody could partly reverse microtubule formation of HUVECs. Previous studies have demonstrated double roles for the WNT signaling in angiogenesis. One possible explanation for this might be that DKK2 may inhibit or induce angiogenesis through directly affect WNT signaling in breast cancer, depending on different Wnt signaling acitivity in different microenvironment. Another possible explanation is that DKK2 could regulate angiogenesis through other pathways such as Notch/Dll4 and VEGF. DKK2 also influence pro-angiogenic factors to regulate angiogenesis. This remains an unmet need for an in-depth study to identify the mechanism of *DKK2*.

## MATERIALS AND METHODS

### Cell lines and tumor samples

Several breast tumor cell lines (BT549, MDA-MB-231, MDAMB-468, MCF-7, T47D, SK-BR-3, YCC-B1, YCC-B3, and ZR-75-1) were used. These cell lines were obtained from the ATCC (American Type Culture Collection, Manassas, VA, USA) or collaborators. All cell lines were maintained in RPMI 1640 media (Gibco-BRL, Karlsruhe, Germany) supplemented with 10% fetal bovine serum (FBS; PAA Laboratories, Linz, Austria), 100 U/mL penicillin, and 100 mg/mL streptomycin at 37°C in a humidified atmosphere containing 5% CO_2_ [32–33]. All tissues were obtained from patients who underwent primary surgery at the Department of Endocrine and Breast Surgery, The First Affiliated Hospital of Chongqing Medical University [34–35]. Clinical and pathological data of all the participants were obtained, and their demographics are summarized in Table 2. This research was approved by the Institutional Ethics Committees of the First Affiliated Hospital of Chongqing Medical University (#20130306), and conformed to the tenets of the Declaration of Helsinki.

**Table 2:** Clinicopathologic features of *DKK2* methylation in breast cancer

Clinicopathological features	Number (n=98)	*DKK2* promoter methylated status		*p* value
		methylated	unmethylated	
**Age**				0.171
≤40	9	6	3	
>40	74	66	8	
unknown	15	13	2	
**grade**				0.731
I	6	6	0	
II	48	42	6	
III	5	4	1	
unknown	39	33	6	
**Tumour size**				0.975
<2.0 cm	15	13	2	
≥2.0 cm ≤5.0cm	63	55	8	
>5.0cm	5	4	1	
unknown	15	13	2	
**Lymph node metastasis**				0.494
Positive	39	32	7	
Negative	44	40	4	
unknown	15	13	2	
**ER status**				0.782
Positive	36	32	4	
Negative	23	19	4	
unknown	39	34	5	
**PR status**				**0.523**
Positive	29	26	3	
Negative	30	25	5	
unknown	39	34	5	
**HR status**				0.313
>+++	46	39	7	
++	10	9	1	
<+	42	37	5	

### DNA and RNA extractions

Genomic DNA was isolated from cell lines and tissues using DNAzol^®^ reagent (Invitrogen, Rockville, MD, USA) and the QIAamp^®^ DNA Mini Kit (Qiagen, Hilden, Germany) according to the manufacturer's protocols. Total RNA was extracted from cell lines and tissues using TRI Reagent® (Molecular Research Center, Cincinnati, OH, USA) [32]. Total DNA and RNA were detected by gel electrophoresis. Samples were stored at −80°C until use.

### 5-aza-deoxycytidine and trichostatin A treatments

5-aza-2′-deoxycytidine (5-aza-dC), a DNA methyltransferase (DNMT) inhibitor, makes DNMT inactivation through DNMT covalent bonding with thiol on cysteine residues, resulting in reactivation genes silenced by promoter methylation. Trichostatin A (TSA), a histone deacetylase inhibitor, plays a significant role in controlling the tightness of DNA around histone. Combination treatment of TSA and 5-Aza-dC results in the synergistic activation of methylated genes.

As previously described [32], breast cancer cell lines were treated with 10 mmol/L 5-aza-2-deoxycytidine (Aza) (Sigma-Aldrich, Steinheim, Germany) for 3 days and further treated with or without 100 nmol/L TSA (Sigma-Aldrich) for an additional 24 h.

### Reverse transcriptase-PCR and real-time PCR

The reverse transcriptase-polymerase chain reaction (RT-PCR) was performed as previously described [32, 36]. *GAPDH* was amplified as a control. Samples were assayed in a 10 μL reaction mixture containing 2 μL of cDNA. The primer sequences are in listed in Table 3. RT-PCR was carried out using Go-Taq (Promega, Madison, WI, USA). Reaction was under the following conditions: 32 cycles for *DKK2* and 23 cycles for *GAPDH*. Real-time PCR was performed according to the manual (HT7500 System; Applied Biosystems), the expression level of *DKK2* in paired surgical margin tissues was considered as baseline.

**Table 3:** List of primers used in this study

PCR	Primer	Sequence (5′-3′)	Product size (bp)	PCR Cycles	Annealing temperature (°C)
MSP	DKK2-M1	AGAGTTAAATCGTCGAGATTTC	146	40	60
	DKK2-M2	CTAAAAACAATCAAATACGAAACG			
	DKK2-U1	GGAGAGTTAAATTGTTGAGATTTT	149	40	58
	DKK2-U2	ACTAAAAACAATCAAATACAAAACA			
RT-PCR	DKK2-F	GTACCAAGGACTGGCATTCG	169	32	55
	DKK2-R	ATCTCGGTGGCAGCGCTTCT			

### Bisulfite treatment and methylation-specific PCR (MSP)

Bisulfite modification of DNA and methylation-specific PCR (MSP) were carried out as previously described [37–38]. The methylation-specific primers are listed in Table 2. MSP analysis revealed no amplified product in any not-bisulfited DNA by MSP primers, thus specific. MSP was performed for 40 cycles using AmpliTaq^®^-Gold DNA Polymerase (Applied Biosystems) with annealing temperatures of 60°C or 58°C. Methylated and non-methylated human DNAs were used as positive and negative controls, respectively. MSP products were identified on 2% agarose gel containing 100 bp DNA markers (MBI Fermentas, Vilnius, Lithuania).

### Construction of LV-*DKK2* and stable cell lines

To construct human *DKK2* lentiviral over-expression vector (LV-*DKK2*), using normal testis cDNA as a template, the *DKK2* fragment was amplified by PCR to construct pEZ-Lv105 shuttle plasmid by directly clone. Shuttle plasmid along with and helper plasmids mixture were co-transfected into 293T cells to package LV-*DKK2*, and the virus titer was determined by real-time PCR and drug screening method, respectively. MDA-MB-231 and MCF7 stable cells infected with LV-*DKK2* were obtained under the pressure of puromycin (2 μg/ml, BioVision, Inc., CA, USA). Total RNA from infected cells was extracted, treated with TURBO™ DNase (Ambion, Austin, TX, USA). Proteins from two cell lines were solubilized and extracted using the Protein Extraction kit (Thermo Scientific, #23225). RT-PCR and western blot were used to measure ectopic expression of *DKK2* prior to the other experiments.

### Colony formation assays

Colony formation assays were performed as previously described [32]. Cells infected by lentivirus were plated in 6-well plates at a density of 1 × 10^3^ cells/well. Surviving colonies (≥ 50 cells/colony) were counted after staining with Gentian violet. All experiments were performed in triplicate.

### Cell proliferation assay

MDA-MB-231 and MCF7 stably cells were cultured in 6-well plates and grown overnight. After 24, 48, and 72 h, proliferation was measured using the Cell Counting Kit-8 (CCK-8; Beyotime, Shanghai, China) [32]. The experiments were independently repeated three times.

### Analyses of the cell cycle and apoptosis

To assess cell cycle status [39], MDA-MB-231 and MCF7 cells were seeded (1 × 10^6^ cells/well) in 6-well plates. After 48 h, cells were collected and centrifuged at 800 rpm for 5 min, then washed with phosphate-buffered saline (PBS) twice, and fixed in ice-cold 70% ethanol for at least 24 h, and treated with 100 μL of 50 mg/L propidium iodide for 30 min at 4°C in the dark. The cell cycle data were analyzed by CELL Quest software (BD Biosciences, San Jose, CA, USA). For apoptosis analyses, acridine orange/ethidium bromide (AO/EB) fluorescence staining was used [39]. Cells were re-plated in 6-well plates. After 24 h, cells were washed in PBS three times then stained with AO/EB for 5 min and visualized immediately under a fluorescence microscope (LEICA CTR4000B; Leica Microsystems, Buffalo Grove, IL, USA). The percentage of apoptotic cells was then calculated by the formula: percentage of apoptotic cell (%) = (amount of apoptotic cells/total cells examined) × 100%.

### Wound healing and Transwell^®^ assays for cell migration

Cell mobility was assessed using a scratch wound healing assay. *DKK2* stably infected cells (MDA-MB-231 and MCF7) were cultured in 6-well plates until confluent. The cell layers were carefully wounded using sterile tips and then cells were washed with PBS, cultured in 5% FBS-RPMI 1640. After incubation for 12, 24, and 48 h, the cells were photographed under a 10× objective lens. Experiments were performed in triplicate. *In vitro* Transwell® assays were carried out as described previously [40].

### Microtube formation and conditioned medium assays

Conditioned media were collected by incubating MCF7 breast cancer cells infected with lentivirus LV-DKK2 or vector alone without serum for 24 h. After Matrigel® culturing, (Corning Life Sciences, Bedford, MA, USA) cells were thawed on ice (approximately 1-2 h). The Matrigel® was added into the 96-well plates (50 μL into each well) that were then incubated at 37°C for 30 min to allow the Matrigel® to polymerize. A total of 1 × 10^4^ human umbilical vein endothelial cells (HUVEC) were seeded into each well and incubated with 100 μL conditioned media from LV-DKK2 or vector alone stably infected breast cancer cells (MCF7). HUVECs were then incubated for 6 h to allow microtubule formation. Image analyses of tube lengths were carried out using Image J software. To eliminate other factors effecting microtubule formation, anti-DKK2 primary antibody was added in the conditioned media. Transwell® assays for HUVECs in condition medium were performed to further determine whether *DKK2* influenced the migration of HUVECs.

The co-culture system was used to detect the influence of DKK2 on HUVEC migration. Briefly, HUVEC cells were seeded at a density of 2 × 10^5^ cells/mL into the upper chamber of a Transwell® (Corning Life Sciences) with inserts containing 8-μm pore size polyethylene terephthalate (PET) membranes (Corning Life Sciences). MDA-MB-231 or MCF7 with LV-*DKK2* or vector was seeded in the lower chamber with RPMI 1640 containing 10% FBS. After a 24h incubation at 37°C, cells remaining in the upper chamber were removed carefully with a cotton swab and the membrane was cut with an operating knife. The side facing the lower chamber was stained with 0.05% crystal violet and attached cells were counted under a light microscope. Each experiment was performed three times.

### Indirect immunofluorescence determinations

MDA-MB-231 and MCF7 cells were seeded (1×10^6^cells/well) in 24-well plates on coverslips and allowed to grow overnight, the cultures were then infected transiently with LV-*DKK2*. Coverslips were stained by indirect immunofluorescence double staining as described previously [40]. Briefly, cells were incubated with primary antibodies against E-cadherin (#1702-1; Epitomics), vimentin (#2707-1; Epitomics), or DKK2 (ab38594; Abcam), and then incubated with Alexa Fluor® 594- (Invitrogen) or FITC-conjugated (Dako, Carpinteria, CA, USA) secondary antibody against mouse or rabbit IgG. Cells were then counterstained with 4′, 6-diamidino-2-phenylindole (DAPI) and imaged with a confocal laser scanning microscope.

### Tissue microarray and immunohistochemistry analyses

To evaluate the expression levels of DKK2 in breast cancer tissues, tissue microarrays (TMA) were constructed using paraffin embedding, including 30 pairs of primary tumors and corresponding tumor margin tissues (Biochip Co., Ltd., Shanghai, China). IHC was performed according to IHC procedure as described previously [32, 41]. Anti-DKK2 was obtained from abcam (ab38594; Abcam). Sections were incubated with primary antibody (1:200 dilution) overnight at 4°C, followed by the secondary antibody (1:2000 dilution) at 37°C for 30 min. Finally, the slides were counterstained with hematoxylin. The mean optical density (MOD) was analyzed using IPP6.0 (Image Pro Plus 6.0, Silver Spring, MD, USA)

### *In vivo* tumor model

Animal experiments were performed to determine whether *DKK2* inhibits tumor growth *in vivo*. Female BALB/c nude mice (aged 4-6 weeks, weighing 18-22 g) were purchased from the Experimental Animal Center of Chongqing Medical University (CQMU), China. This study was approved by ethics committee of CQMU. Stable *DKK2*-expressing MDA-MB-231 cells or control cells (3 × 10^6^ cells in 0.1 mL PBS) were injected into the backs of female nude mice (n=6). Tumor diameter was measured every 3 days for 30 days. Tumor volume (mm^3^) = length × width^2^ × 0.52.

### Terminal deoxynucleotidyl transferase (TUNEL) analyses

TUNEL assay was used to detect apoptotic cells in tumor xenograft tissues following our previously described procedures [41]. TUNEL apoptosis detection kit was provided by Roche (Roche Applied Science, Pleasanton, CA, USA). Images were captured by Leica LSM 400 laser scanning microscope (Leica), and the rate of apoptosis was quantified using Image Pro Plus software (Media Cybernetics).

### Western blot

Western blot was performed as described previously [34]. A total of 40μg of protein lysates were separated by sodium dodecyl sulphate/polyacrylamide gel electrophoresis (SDS-PAGE) and then transferred onto a polyvinylidene difluoride (PVDF) membrane (Bio-Rad, Hercules, CA, USA). The primary antibodies were against: DKK2 (ab38594, Abcam), active β-catenin (#05-665; Merck Millipore, Billerica, MD, USA), total β-catenin (#2677; Cell Signaling Technology, Danvers, MA, USA), c-Myc (#1472-1; Epitomics, Cambridge, MA, USA), cyclin D1 (#1677-1; Epitomics), occludin (ab31721; Abcam), vimentin (#2707-1; Epitomics), Ecad (#1702-1; Epitomics), N-cadherin (ab98952;Abcam), and β-actin (LK-ab008-100; Liankebio, China), and GAPDH (#AE082046; Beijing Biosynthesis Biotechnology, Beijing, China) was used as a control. Proteins were visualized using an enhanced chemiluminescence kit (Amersham Pharmacia Biotech, Piscataway, NJ, USA).

### Statistical analyses

Statistical analyses were performed with SPSS software, version16 (SPSS, Chicago, IL, USA). Student's *t*-test, the x^2^ test, and Fisher's exact test were used to compare methylation status and clinicopathological parameters [39]. For all tests, *p* < 0.05 was considered statistically significant.

## CONCLUSION

All in all, our data demonstrated that *DKK2* can inhibit breast carcinoma growth via suppressing Wnt/β-catenin signaling. Furthermore, the *DKK2* promoter methylation status could be a potential tumor marker for early found of mammary carcinoma, or even offer a new therapeutic method for breast carcinoma.

## Abbreviations

DKK2: Dickkopf-related protein 2
LRP: LDL receptor-related proteins
MSP: methylation-specific PCR
AO/EB: acridine orange/ethidium bromide
HUVECs: human umbilical vein endothelial cells
EC: endothelial cells
TUNEL: Terminal deoxynucleotidyl transferase
Aza: 5-aza-2′-deoxycytidine
TSA: Trichostatin A
EMT: epithelial-mesenchymal transition
